# Paracrine regulation of matrix metalloproteinases contributes to cancer cell invasion by hepatocellular carcinoma-secreted 14-3-3σ

**DOI:** 10.18632/oncotarget.9234

**Published:** 2016-05-09

**Authors:** Chia-Chia Liu, Tzu-Ching Chang, Yi-Ting Lin, Yen-Ling Yu, Bor-Sheng Ko, Li-Ying Sung, Jun-Yang Liou

**Affiliations:** ^1^ Institute of Cellular and System Medicine, National Health Research Institutes, Zhunan 350, Taiwan; ^2^ Institute of Biotechnology, National Taiwan University, Taipei 106, Taiwan; ^3^ Graduate Institute of Clinical Medical Science, China Medical University, Taichung 404, Taiwan; ^4^ Metabolomic Medicine Research Center, China Medical University, Taichung 404, Taiwan; ^5^ Institute of Structural Biology, National Tsing Hua University, Hsinchu 300, Taiwan; ^6^ Department of Internal Medicine, National Taiwan University Hospital, Taipei 100, Taiwan; ^7^ Graduate Institute of Basic Medical Science, China Medical University, Taichung 404, Taiwan; ^8^ PhD. Program for Aging, China Medical University, Taichung 404, Taiwan

**Keywords:** 14-3-3σ, hepatocellular carcinoma, invasion, matrix metalloproteinase, paracrine

## Abstract

14-3-3σ overexpression results in enhanced hepatocellular carcinoma (HCC) cell migration and HCC tumor vascular-invasion is significantly associated with 14-3-3σ expression. However, increased expression of 14-3-3σ paradoxically suppresses *in vitro* cell invasion of HCC. We hypothesize that surrounding tumor-associated stromal cells play a crucial role in 14-3-3σ-regulated HCC cell invasion. In this study, H68 fibroblasts, THP-1 and phorbol-12-myristate-13-acetate (PMA)-treated THP-1 (PMA-THP-1) cells were incubated with conditioned media of control (control-CM) and 14-3-3σ-overepxressing cells (14-3-3σ-CM), followed by co-culture with HCC cells. Invasiveness of HCC cells was examined by a Boyden chamber assay. HCC cells co-cultured with 14-3-3σ-CM treated cells significantly enhanced their invasive ability compared with control-CM treated cells. Moreover, incubation with 14-3-3σ-CM induced differential expression profiles of matrix metalloproteinases (MMPs) in fibroblasts (MMP-1, MMP-2, MMP-9, MMP-12 and MMP-14), THP-1 (MMP-1 and MMP-12) and PMA-THP-1 cells (MMP-2, MMP-12 and MMP-14). In contrast, silencing of 14-3-3σ by siRNA significantly abolished 14-3-3σ-CM induced MMPs. In addition, treatment with recombinant 14-3-3σ (r14-3-3σ) protein exhibits a similar expression profile of MMPs induced by 14-3-3σ-CM in fibroblasts, THP-1 and PMA-THP-1 cells. Finally, knockdown of aminopeptidase N (APN) significantly abrogated r14-3-3σ induced expression of MMPs in HS68 fibroblasts. These results suggest that HCC-secreted 14-3-3σ promotes expression of MMPs in cancerous surrounding cells *via* an APN dependent mechanism. 14-3-3σ has a paracrine effect in educating stromal cells in tumor-associated microenvironment.

## INTRODUCTION

The tumor microenvironment is comprised of multiple cell types including cancer cells, carcinoma-associated fibroblasts, invading inflammatory cells, and endothelial cells [1–4]. Cancer cells interact and associate with surrounding stromal cells and subsequently “educate” stromal cells to induce tumor growth, invasion as well as metastasis [5–9]. Thus, interplay and synergism of cancer cells with tumor-associated stromal cells are crucial in promoting tumor progression.

14-3-3 proteins have been implicated in regulating tumor progression of hepatocellular carcinoma (HCC) [10–16]. Several recent studies have indicated that expression of 14-3-3β, 14-3-3ε, 14-3-3γ, 14-3-3σ and 14-3-3ζ proteins are elevated in HCC [10–16]. Moreover, increased expression of 14-3-3 proteins is correlated with high risk of metastasis and worse overall HCC survival rate [10–13]. 14-3-3 proteins are therefore considered novel prognostic biomarkers and therapeutic targets of HCC. Among these 14-3-3 isoforms, the expression of 14-3-3σ in HCC tumors is controversial. An earlier study has indicated that expression of 14-3-3σ is eliminated by frequent hypermethylayion of CpG islands on the promoter region in HCC [17]. In contrast, recent studies have shown that 14-3-3σ is overexpressed in HCC [15–16, 18]. It was reported that increased expression of 14-3-3σ promotes HCC cell migration *via* the induction of heat shock protein 70 (Hsp70) and expression of 14-3-3σ is associated with HCC vascular-invasion [15]. Unexpectedly, increased expression of 14-3-3σ paradoxically suppresses cell invasion of HCC [15]. These results indicate that the regulating processes of 14-3-3σ in HCC cell migration/invasion and tumor metastasis are complicated and other essential synergistic regulators are probably involved. In addition, it has been shown that keratinocyte-secreted 14-3-3σ affects muscle remodeling by upregulation of matrix metalloproteinases 1 (MMP-1) in keratinocyte associated fibroblasts [19–22]. Keratinocyte-released 14-3-3σ induced MMP-1 expression through the activation of *c-fos* and MAPK pathway in keratinocyte-associated fibroblasts [21]. Moreover, aminopeptidase N (APN/CD13) was identified as a potential fibroblast receptor for secreted 14-3-3σ and consequently stimulated MMP-1 expression in keratinocyte associated fibroblasts [22]. However, whether paracrine effect of 14-3-3σ-APN machinery involved in regulating tumor progression of HCC remains unclear.

MMPs are a group of endopeptidases that are important in the degradation of the extracellular matrix thus influencing distinct cellular functions [23–25]. MMPs contribute to the regulation of cancer cell invasion and tumor metastasis [26–30]. Expression of various MMPs including MMP-1, MMP-2, MMP-9, MMP-12 and MMP-14 were implicated in regulating HCC tumor progression and prognosis [31–40]. In this study, we found that HCC-secreted 14-3-3σ stimulates MMP expression in cancer-associated cells. Co-culturing of 14-3-3σ conditioned medium (14-3-3σ-CM)-incubated fibroblasts, monocytes and macrophages with HCC cells resulted in promoting cancer cell invasion. Thus, we hypothesize that a potential paracrine regulation of MMPs may contribute to promote cancer cell invasion by HCC-secreted 14-3-3σ.

## RESULTS

### HCC invasiveness is enhanced by co-culturing with 14-3-3σ-CM incubated cells

**Figure 1:**
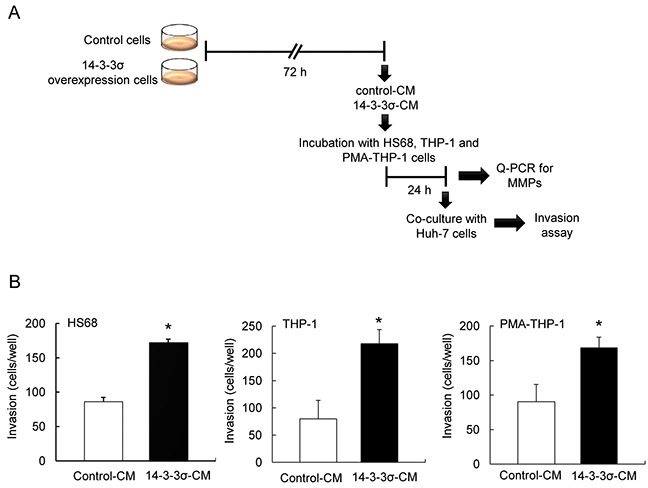
Co-culture of 14-3-3σ-CM treated stromal cells enhances cell invasion of Huh-7 cells **A.** A flow chart of the experimental design. **B.** CM of control and 14-3-3σ overexpression cells was harvested. Subsequently, HS68, THP-1 and PMA-THP-1 cells (1 × 10^4^) were incubated with control-CM or 14-3-3σ-CM, followed by co-cultured parental Huh-7 cells (1 × 10^5^). Cell invasion was determined by a Boyden chamber assay. These results are from three independent experiments. Scale bars: mean ± SD. *, *P*<0.05.

Our earlier study has indicated that overexpression of 14-3-3σ significantly correlates with vascular-invasion of HCC tumors [15]. However, 14-3-3σ overexpression induces cell migration [15] but paradoxically reduces *in vitro* cell invasion of HCC (Supplementary Figure S1). We hypothesized that 14-3-3σ may promote HCC invasion *via* regulating and educating tumor associated stromal cells. To test this hypothesis, Huh-7 cells were transfected with 14-3-3σ overexpression and control vectors, followed by selection to establish stable cells [15]. The expression of 14-3-3σ was confirmed in stable cells (control *vs*. 14-3-3σ) by Western blot analysis (Supplementary Figure S2). We next used HS68 cells (fibroblasts), THP-1 (monocytes) and PMA-THP-1 (phorbol-12-myristate-13-acetate-treated THP-1, macrophages) as model cells to test whether HCC-secreted 14-3-3σ contributes to the association with or education of stromal cells. Control-CM and 14-3-3σ-CM (cultured for 72 h) were harvested (experimental procedures illustrated in Figure 1A). Subsequently, HS68, THP-1 and PMA-THP-1 cells were incubated with control-CM or 14-3-3σ-CM for 24 h, followed by co-culturing with fluorescence-labeled parental Huh-7 cells in the upper wells of Boyden chambers. Cell invasion ability was further examined by the trans-well system. We found that co-culturing 14-3-3σ-CM-incubated HS68, THP-1 and PMA-THP-1 significantly augmented Huh-7 cell invasion (Figure 1B).

### Induced expression of MMPs in 14-3-3σ-CM incubated cells

**Figure 2:**
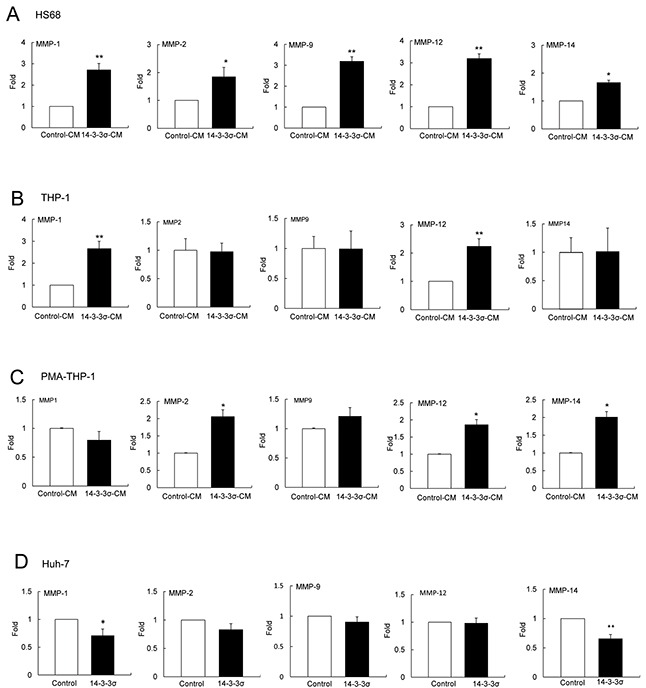
14-3-3σ-CM induces expression of MMPs in stromal cells HS68, THP-1 and PMA-THP-1 cell were incubated with control-CM and 14-3-3σ-CM for 24 h and expression of MMPs was determined by Q-PCR. **A.** Expression of MMP-1, MMP-2, MMP-9, MMP-12 and MMP-14 in HS68 cells. **B.** Expression of MMP-1, MMP-2, MMP-9, MMP-12 and MMP-14 in THP-1 cells. **C.** Expression of MMP-1, MMP-2, MMP-9, MMP-12 and MMP-14 in PMA-THP-1 cells. **D.** Expression of MMP-1, MMP-2, MMP-9, MMP-12 and MMP-14 in 14-3-3σ overexpression compared to control Huh-7 cells. These results are from three independent experiments. Scale bars: mean ± SD. *, *P*<0.05, **, *P*<0.01.

Expression of MMPs participates in cancer cell invasion and tumor metastasis [26–30]. To investigate whether 14-3-3σ-CM induces MMPs in stromal cells, we incubated HS68, THP-1 and PMA-THP-1 cells with control-CM and 14-3-3σ-CM for 24 h (Figure 1A). Expression of MMPs including MMP-1, MMP-2, MMP-9, MMP-12 and MMP-14 was determined by Q-PCR (Real-time Quantitative PCR). 14-3-3σ-CM significantly induced MMP-1, MMP-2, MMP-9, MMP-12 and MMP-14 in HS68 cells (Figure 2A). Additionally, 14-3-3σ-CM stimulated MMP-1 and MMP-12 expression in THP-1 cells (Figure 2B), as well as MMP-2, MMP-12 and MMP-14 expression in PMA-THP-1 cells (Figure 2C). However, 14-3-3σ-CM did not influence the expression of MMP-2, MMP-9 and MMP-14 in THP-1 cells (Figure 2B), as well as MMP-1 and MMP-9 in PMA-THP-1 cells (Figure 2C). Moreover, we have examined MMPs expression in 14-3-3σ overexpression and control Huh-7 cells. Expressions of MMP-1 and MMP-14 were decreased but MMP-2, MMP-9 and MMP-12 had no significant difference in 14-3-3σ overexpression cells compared with control cells (Figure 2D).

**Figure 3:**
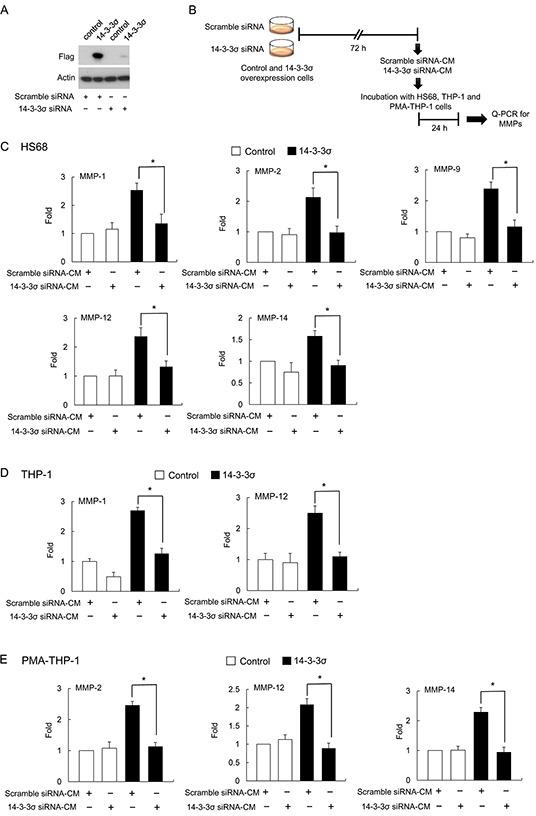
14-3-3σ silencing abolished 14-3-3σ-CM induced MMPs expression **A.** Efficacy of 14-3-3σ silencing by siRNA was confirmed by Western blot analysis. **B.** A flow chart of the experimental design. **C.** HS68 fibroblasts were incubated with CM derived from 14-3-3σ silencing with siRNA or scramble siRNA in 14-3-3σ stable and control cells. Expression of MMPs was determined by Q-PCR. **D.** THP-1 cells were incubated with CM and expression of MMPs was determined by Q-PCR. **E.** PMA-THP-1 cells were incubated with CM and expression of MMPs was determined by Q-PCR. These results are from three independent experiments. Scale bars: mean ± SD. *, *P*<0.05.

To further confirm the involvement of 14-3-3σ in regulating MMP expression in a paracrine manner, we next employed the knockdown of 14-3-3σ. Stable cells of control and 14-3-3σ were transfected with scramble and 14-3-3σ siRNA and expression was validated by Western blot analysis (Figure 3A). CMs were harvested and subsequently applied for incubation with HS68, THP-1 and PMA-THP-1 cells and expression of MMPs was determined by Q-PCR (Figure 3B). Incubation with CM of 14-3-3σ silencing cells (14-3-3σ siRNA-CM) significantly attenuated the expression of MMP-1, MMP-2, MMP-9, MMP-12 and MMP-14 in HS68 cells (Figure 3C); expression of MMP-1 and MMP-12 in THP-1 cells (Figure 3D); and expression MMP-2, MMP-12 and MMP-14 in PMA-THP-1 cells as compared with scramble siRNA-CM (Figure 3E). In addition, 14-3-3σ siRNA-CM had no significant effect on the expression of MMP-2, MMP-9 and MMP-14 in THP-1 cells (Supplementary Figure S3A) and MMP-1 and MMP-9 in PMA-THP-1 cells (Supplementary Figure S3B).

### 14-3-3σ participates in regulating expression of MMPs

**Figure 4:**
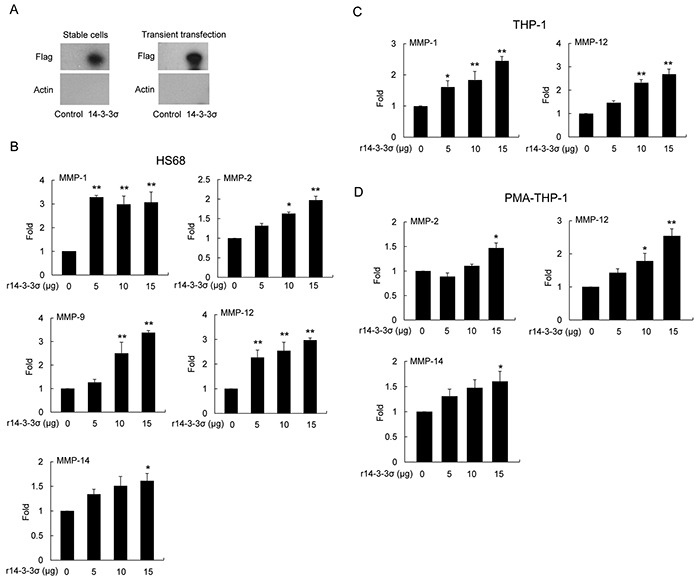
Induced expression of MMPs in stromal cells by 14-3-3σ recombinant protein **A.** Expression of 14-3-3σ in CM from cells of stable 14-3-3σ overexpression and transient transfection was determined by Western blot analysis. **B.** HS68 **C.** THP-1 and **D.** PMA-THP-1 cells were treated with different concentrations of recombinant 14-3-3σ (r14-3-3σ) protein for 24 h. Expression of MMPs was determined by Q-PCR. These results are from three independent experiments. Scale bars: mean ± SD. *, *P*<0.05, **, *P*<0.01.

It has been reported that keratinocyte-releasable 14-3-3σ stimulates MMP-1 expression in dermal fibroblasts [19–22]. We investigated whether HCC-secreted 14-3-3σ directly participates in modulating MMP expression of HS68, THP-1 and PMA-THP-1 cells. We first confirmed that 14-3-3σ is released from 14-3-3σ-overexpressing HCC cells. Expression of 14-3-3σ was detected in the both 14-3-3σ-CM (stable cells) and 14-3-3σ transient transfection cells by Western blot analysis of the flag tag (Figure 4A). Knockdown of 14-3-3σ by siRNAs significantly reduced the secreted 14-3-3σ in the CM (Supplementary Figure S4). To further investigate the role of 14-3-3σ in educating tumor-associated cells, we amplified and purified the recombinant 14-3-3σ (r14-3-3σ) protein (Supplementary Figure S5A) and subsequently confirmed by Western blot analysis of 14-3-3σ (Supplementary Figure S5B). HS68, THP-1 and PMA-THP-1 cells were directly treated with different concentrations of r14-3-3σ (0-15 μg/ml) for 24 h. We found that treatment of r14-3-3σ induced expression of MMPs in HS68 (Figure 4B), THP-1 (Figure 4C) and PMA-THP-1 cells (Figure 4D) with similar patterns to 14-3-3σ-CM.

### 14-3-3σ induced expression of MMPs in associated cells occurs *via* an APN dependent mechanism

**Figure 5:**
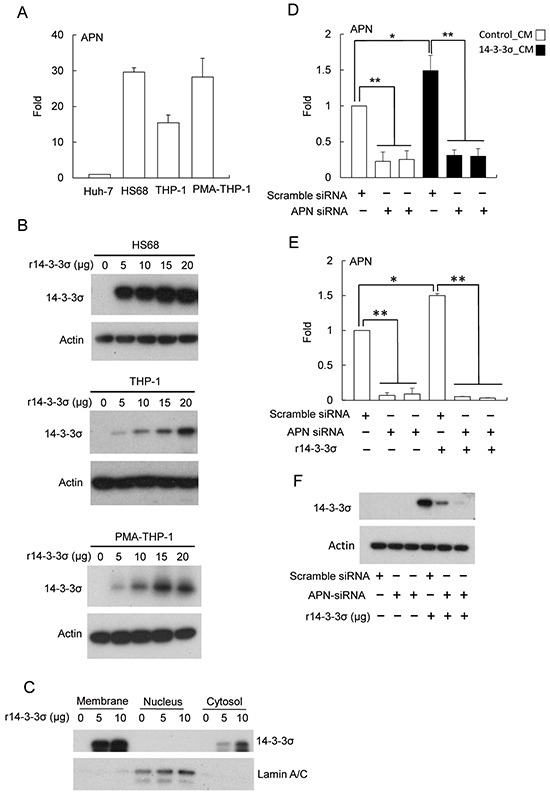
The role of APN for uptake of 14-3-3σ in fibroblasts **A.** Endogenous APN expression levels in Huh-7, HS68, THP-1 and PMA-THP-1 cells were determined by Q-PCR. **B.** HS68, THP-1 and PMA-THP-1 cells were treated with different concentrations (0-20 μg/ml) of r14-3-3σ for 24 h and the levels of 14-3-3σ were determined by Western blot analysis. Actin was used as a loading control. **C.** 14-3-3σ in subcellular fractions of membrane, nuclei and cytosol were determined by Western blot analysis. Lamin A/C was used as nuclear loading control. **D.** Silencing of APN by siRNA (*vs.* scramble siRNA) treated with control-CM and 14-3-3σ-CM or **E.** r14-3-3σ protein was determined by Q-PCR. **F.** Knockdown of APN with siRNA abolished the uptake of r14-3-3σ in HS68 cells was determined by Western blot analysis. Actin was used as a loading control. These results are from three independent experiments. Scale bars: mean ± SD. *, *P*<0.05, **, *P*<0.01.

14-3-3σ regulates MMP-1 expression of dermal fibroblasts *via* associating with cell surface APN [22]. We next studied whether APN is involved in HCC-secreted 14-3-3σ induced expression of MMPs in stromal cells. We first examined the expression level of APN by Q-PCR. HS68 and PMA-THP-1 cells most abundantly express APN, followed by THP-1 with Huh-7 expressing relatively low amounts of APN (Figure 5A). Since APN is a potential surface receptor for 14-3-3σ [22], we investigated whether 14-3-3σ is detectable in r14-3-3σ-treated stromal cells. HS68, THP-1 and PMA-THP-1 cells were incubated with different concentration of r14-3-3σ (0-20 μg/ml) for 24 h. Cells were harvested and 14-3-3σ levels were determined by Western blot analysis. 14-3-3σ can be detected in r14-3-3σ-treated HS68, THP-1 and PMA-THP-1 cells (Figure 5B). We next examined the levels of r14-3-3σ in membrane, nuclear and cytosolic fractions of HS68 cells. We found that r14-3-3σ was abundantly accumulated in membrane and partially located in the cytosolic fractions (Figure 5C). To further investigate whether APN is involved in uptake of r14-3-3σ into stromal cells, HS68 cells were then transfected with APN siRNA followed by incubation with 14-3-3σ CM/control CM or r14-3-3σ. APN siRNA significantly suppressed APN expression although 14-3-3σ-CM and r14-3-3σ slightly induced APN expression (Figure 5D and 5E). The protein level of 14-3-3σ transfected with APN siRNAs was significant reduced when compared with the scramble siRNA control in HS68 cells (Figure 5F).

**Figure 6:**
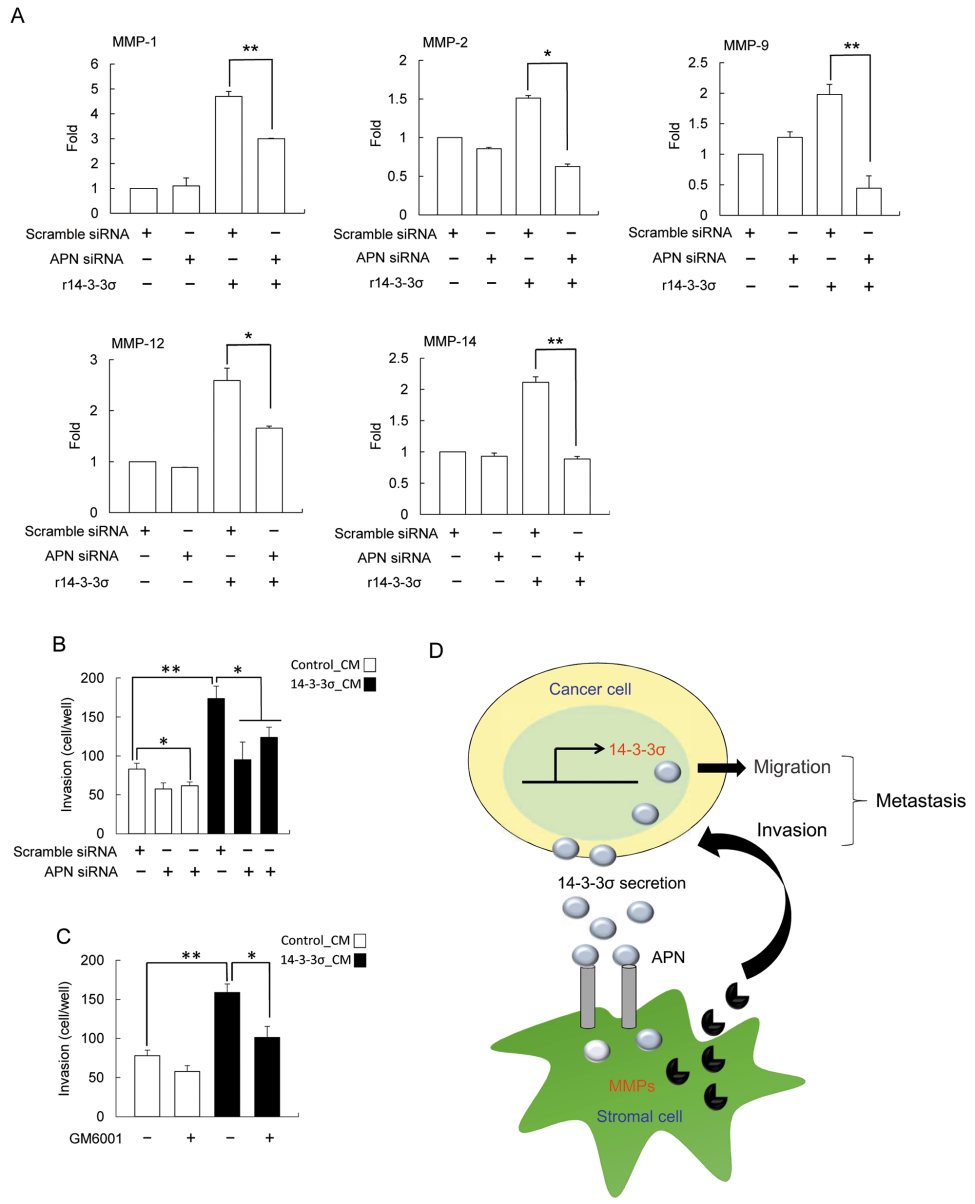
Silencing of APN abrogated the effect of 14-3-3σ induced MMPs expression **A.** HS68 cells were transfected with APN and scramble siRNAs for 24 h, followed by treatment with r14-3-3σ (10 μg/ml) for an additional 24 h. Expression of MMPs (MMP-1, MMP-2, MMP-9, MMP-12 and MMP-14) were determined by Q-PCR. **B.** and **C.** HS68 cells were transfected with scramble/APN siRNA or treated with 30 μM GM6001 followed by incubation with control-CM or 14-3-3σ-CM. These cells were subsequently co-cultured with Huh-7 cells and cell invasion of Huh-7 cells was determined by trans-well assay. These results are from three independent experiments. Scale bars: mean ± SD. *, *P*<0.05, **, *P*<0.01. **D.** An illustrated scheme for the role of HCC-secreted 14-3-3σ in regulating HCC tumor metastasis.

We next examined whether APN knockdown influences expression of MMPs in HS68 fibroblasts. Silencing of APN resulted in abrogating r14-3-3σ-stimulated expression of MMP-1, MMP-2, MMP-9, MMP-12 and MMP-14 in HS68 fibroblasts (Figure 6A). Finally, HS68 cells transfected with APN siRNA or treated with GM6001 (an inhibitor of MMPs) followed by determination of cell invasiveness with co-cultured of Huh-7 cells. APN siRNA and MMPs inhibitor significantly abolished 14-3-3σ-CM-induced cell invasion (Figure 6B and 6C).

## DISCUSSION

We have shown the paracrine role of HCC-secreted 14-3-3σ in modulating cancer cell invasion *via* stimulating MMP expression in carcinoma associated stromal cells. We have provided evidence that HCC-secreted 14-3-3σ educates stromal cells in the tumor microenvironment. Tumor associated stromal cells play critical role for tumor progression [5–9]. In HCC, an earlier study has demonstrated that depleting tumor-associated macrophages significantly enhances the therapeutic effect of sorafenib by suppressing metastasis and angiogenesis in an *in vivo* xenograft nude mice model [41]. This result affirms the role of tumor associate stromal cells in promoting tumor metastasis and growth. In this work, we found that incubation of HS68, THP-1 and PMA-THP-1 cells with 14-3-3σ-CM or r14-3-3σ induced expression of different MMP expression. Since the expression levels of 14-3-3σ in HS68, THP-1 and PMA-THP-1 are barely detectable (Supplementary Figure S6), it is reasonable to assume that these cells uptake 14-3-3σ secreted from HCC (Figure 6D). In this study, we found that increased HCC-secreted 14-3-3σ results in inducing expression of MMPs in stromal cells *via* an APN-dependent mechanism. However, the molecular mechanism of differential expression of 14-3-3σ in HCC and associated stromal cells needs further investigation.

We have examined the secreted 14-3-3σ level in 14-3-3σ-CM and control-CM by Western blotting analysis. The estimated protein level of 14-3-3σ is approximately 0.15 μg/ml in 14-3-3σ-CM whereas almost undetectable in control-CM (Supplementary Figure S7). Intriguingly, the concentration of 14-3-3σ in 14-3-3σ-CM is relatively lower than r14-3-3σ (5-20 μg/ml) used in treatment with stromal cells for induction of MMPs (Figure 4B-4D). We postulate that post-translational modification of secreted-14-3-3σ or hetero-dimerization in CM may influence the efficacy of the induction of MMPs in stromal cells. In addition, we found that 14-3-3σ-CM induced differential MMPs expression levels in HS68, THP-1 and PMA-THP-1 cells (Figure 2A–2C). In contrast, 14-3-3σ overexpression had no effect on MMP-2, MMP-9 and MMP-12 as well as reduced MMP-1 and MMP-14 expression in Huh-7 cells (Figure 2D). These results imply that endogenous and secreted 14-3-3σ from HCC may regulate distinct cellular functions via differential signaling or mechanism. Further investigation is needed to clarify these possibilities.

Expression of 14-3-3σ is silenced in about 80% of breast tumors but highly expressed in correlation with malignant phenotype of basal-like breast cancer [42]. Expression of 14-3-3σ is associated with poor prognostic outcomes in basal-like subtypes of breast cancer. This indicates that 14-3-3σ contributes to a more aggressive tumor progression [42]. 14-3-3σ is overexpressed in 77.1% of HCC tumors [15]. Although 14-3-3σ does not play as an independent prognostic factor [15], it was suggested that 14-3-3σ combined with EZH2 significantly correlates with worse clinical HCC outcomes [16]. Here, we demonstrate that an increase of HCC-secreted 14-3-3σ results in synergizing stromal cells and contributes to tumor invasion. Taken together, it is likely that 14-3-3σ overexpression associate with more aggressive or highly invasive cancer cells of HCC by paracrine regulation.

Elevated MMP-1, MMP-2, MMP-9, MMP-12 and MMP-14 expression are correlated with clinical significance of HCC [31–40]. Intriguingly, moderate levels of membrane type 1-MMP and MMP-9 were detected in cancer and stromal cells. However, MMP-2 is predominantly expressed by the tumor stroma in hepatocellular and pancreatic adenocarcinoma [34]. These results, revealing a differential expression profile of MMPs by cancer and stromal cells, are vital in promoting HCC tumor progression. How HCC-secreted 14-3-3σ induces differential profile of MMPs in different stromal cells needs further investigation.

An earlier study reported that 14-3-3σ associates with APN to regulate MMP-1 expression in keratinocyte-associated fibroblasts [22]. We found the endogenous levels of APN in HS68, THP-1 and PMA-THP-1 cells are more abundant than in Huh-7 cells (Figure 5A). The results suggest that the stimulatory machinery responding to 14-3-3σ-APN signaling exists in tumor-associated stromal cells. In addition, we found that r14-3-3σ can be delivered into HS68, THP-1 and PMA-THP-1 cells (Figure 5B), wher eas silencing of APN abolished the delivery of r14-3-3σ (Figure 5F). Thus, the 14-3-3σ-APN interaction may not only induce MMP expression but also mediate 14-3-3σ uptake in stromal cells.

Taken together, in this study, we provided evidence that carcinoma associated stromal cells play important roles in 14-3-3σ-promoting tumor progression. 14-3-3σ may synergize with other signals from its micro-environment milieu and associate with tumor-associated stromal cells in regulating HCC tumor progression.

## MATERIALS AND METHODS

### Cell culture and preparation of conditional medium

HS68 and Huh-7 cells were maintained in Dulbecco's modified Eagle's medium (DMEM, Gibco, Grand Island, NY, USA), and THP-1 cells were cultured in Roswell Park Memorial Institute-1640 (RPMI, Lonza, Walkersville, MD, USA) supplemented with 10% fetal bovine serum (FBS, Hyclone, Logan, UT, USA), 100 U/mL penicillin and 100 U/mL streptomycin in a humidified incubator with 5% CO_2_ at 37°C. 14-3-3σ overexpression stable cells were established as previously described [15]. Briefly, Huh-7 cells were transfected with vectors of p3XFlag-CMV (control) and p3XFlag-14-3-3σ (14-3-3σ) using the PolyJet™ transfection reagent (SignaGen Laboratories, Rockville, MD, USA). The transfected cells were screened by using 500 μg/mL G418 (Biochrom AG, Berlin, Germany) for four weeks. Single colonies of control and 14-3-3σ stable clones (at least 3 in each cell line) were selected and maintained in DMEM with 10% FBS and 200 μg/mL G418. To generate THP-1-derived macrophages, THP-1 cells were treated with 100 nM phorbol-12-myristate-13-acetate (PMA, Sigma-Aldrich, St. Louis, MO) for 24 h [43–45].

To prepare 14-3-3σ-CM and control conditioned media (control-CM), the supernatants of 14-3-3σ overexpression and control stable cells were harvested and centrifuged at 300 g for 10min. The supernatants were transfer to Amicon Ultra-15 filter devices (Millipore, Billerica, MA, USA) followed by centrifugation at 3,000 g for 15min.

### Invasion assay

The cell invasion assay was performed by a modified Boyden chamber system with Bio-coat FluoroBlock cell culture insert (BD Biosciences, San Jose, CA, USA) coated with 0.1% matrigel containing 0.1% bovine serum albumin (BSA)-DMEM medium in the upper chambers. DMEM medium with 100 μg/mL fibronectin (BD Biosciences), epidermal growth factor (80 ng/mL), and 10% BSA were added to the bottom chambers. Parental Huh-7 cells were trypsinized and re-suspended, followed by incubation with pre-warmed CellTracker dye (CellTracker™, Invitrogen Life Technologies, Grand Island, NY, USA) for 45 minutes. 1 × 10^5^ of CellTracker-stained Huh-7 cells combined with 1 × 10^4^ of CM-incubated HS68, THP-1 or PMA-THP-1 cells were co-cultured in the upper chambers for 16 h. For MMP inhibitor experiment, HS68 cells were treated 30 μM GM6001 (Millipore, Billerica, MA, USA) for 1hr. The invasive cells were fixed with 2% formaldehyde for 20 minutes and efficiency of invasiveness was determined by fluorescence microscope with emission wavelength at 490 nm.

### Western blot analysis

Cells were lysed with ice cold RIPA (Radioimmunoprecipitation assay) buffer (0.5 mol/L Tris-HCl, pH 7.4, 1.5 mol/L NaCl, 2.5% deoxycholic acid, 10% NP-40 (nonyl phenoxypolyethoxylethanol), 10 mM/L EDTA; Millipore) containing a protease inhibitor cocktail (Roche, Basel, Switzerland). Cell lysates were centrifuged at 16,100g at 4°C for 20 minutes. Protein concentrations were determined and 20 μg of total proteins were applied to the gradient SDS-PAGE (sodium dodecyl sulfate polyacrylamide gel electrophoresis) gel and immunoblotted onto PVDF (Polyvinylidene difluoride) membranes. The membranes were blocked, incubated with primary antibodies against Flag or actin (Sigma-Aldrich), 14-3-3σ (Abcam PLC, Cambridge, UK) followed by an incubation with a secondary antibody conjugated horseradish-peroxidase in PBST. Protein levels were determined by the use of enhanced chemiluminescence reagents.

### Quantitative real-time PCR (Q-PCR)

Total RNA was extracted by use of the RNAspin Mini Kit (QIAGEN, Valencia, CA, USA) and cDNA was synthesized from 2 to 5 μg RNA by use of the random primers and SuperScript™ III Reverse Transcriptase cDNA Synthesis Kit (Invitrogen™ Life Technology). Quantitative real-time PCR using SYBR Green (Kapabiosystem, Wilmington, MA, USA) with specific oligonucleotide primers of MMP-1, MMP-2, MMP-9, MMP-12, MMP-14 and aminopeptidase N (APN) (Supplementary Table S1) were detected by the AB 7900HT system (Applied Biosystems, Foster city, CA, USA). Applied Biosystems Relative Quantification Manager Software version 1.2 was used to analyze the relative gene expression in each sample by the comparative cycle threshold (Ct) method. Gene expression was normalized to that of glyceraldehyde-3-phosphate dehydrogenase.

### Transfection of siRNAs

Silencing of 14-3-3σ and APN with siRNA (small interfering RNA) was purchased from Invitrogen™ (Supplementary Table S2), including scramble siRNA (Cat.No.12935-112). Transfections of siRNAs were performed by using Lipofectamine™ RNAiMAX (Invitrogen™ Life Technology) and harvested at the indicated time for further analysis.

### Recombinant protein expression

The human full-length 14-3-3σ cDNA was subcloned into the prokaryotic expression vector pET28a (+) (Addgene, Cambridge, MA) for expressing a fusion protein tagged with histidine (His). The N-terminal His_6_-tagged 14-3-3σ vectors were transformed into BL-21 (DE3) bacteria (Yeastern Biotech Co., Taipei, Taiwan) and grown at 37°C in LB medium with 50μg/mL kanamycin followed by induction with 1 mM isopropyl-β-D-thiogalactoside (IPTG). His-tagged 14-3-3σ was amplified and purified by using a Ni-Ten resin according to the manufacturer's instructions (Macherey-Nagel GmbH & Co. KG, Düren, Germany).

### Subcellular fractionation

HS68 cells were treated with 5 or 10 μg/ml r14-3-3σ for 1hr. Cells were harvested and the subcellular fractions of membrane, nuclei and cytosol were prepared by ProteoExtract subcellular proteome extraction kit (Calbiochem, San Diego, CA, USA).

### Statistical analysis

A Student's *t*-test was used to analyze differences between the two experimental groups. A *P* value <0.05 was considered statistically significant.

## SUPPLEMENTARY FIGURES AND TABLES


