# Supplementary material for: Paracrine regulation of matrix metalloproteinases contributes to cancer cell invasion by hepatocellular carcinoma-secreted 14-3-3σ
Source: Oncotarget. 2016 May 9;7(24):36988–99. doi: 10.18632/oncotarget.9234 (PMC5095053; doi:10.18632/oncotarget.9234)
Supplement: Supplementary file 1 [file oncotarget-07-36988-s001.pdf]

Paracrine regulation of matrix metalloproteinases contributes to cancer cell invasion by hepatocellular carcinoma-secreted 14-3-3σ

SUPPLEMENTARY FIGURES AND TABLES

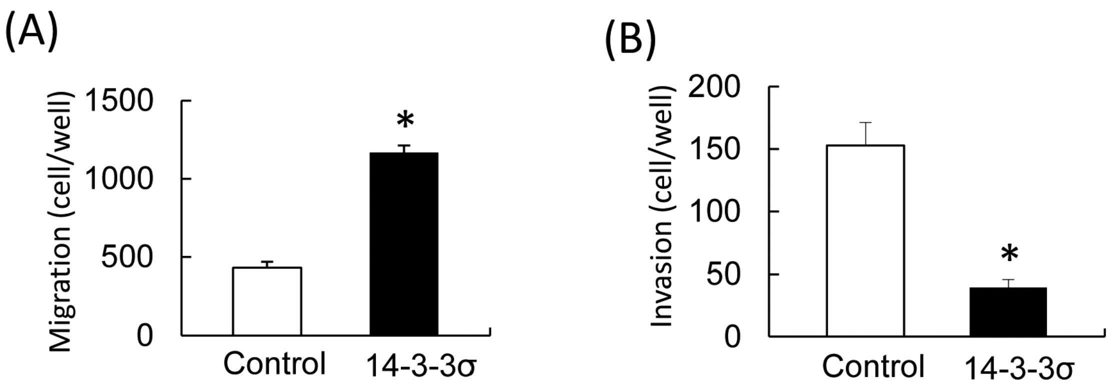

Supplementary Figure S1: Effect of 14-3-3σ overexpression on cell migration and invasion of HCC cells. A. Overexpression of 14-3-3σ promotes cell migration. **B.** Overexpression of 14-3-3σ reduces cell invasion. Efficacy of cell migration or invasion was examined by a two-chamber analysis. These results are from three independent experiments. Scale bars: mean ± SD. *, *P*<0.05, **, *P*<0.01.

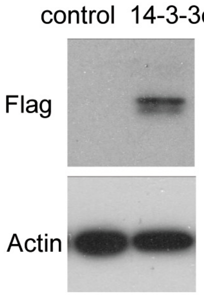

Supplementary Figure S2: Expression of 14-3-3σ in stable Huh-7 cells. Huh-7 cells were transfected with p3XFlag-CMV (control) and Flag-tagged 14-3-3σ overexpression vectors, followed by selection with G418 for 4 weeks. Expression of 14-3-3σ in stable cells was confirmed by Western blot analysis of flag antibody. Actin was used as loading control.

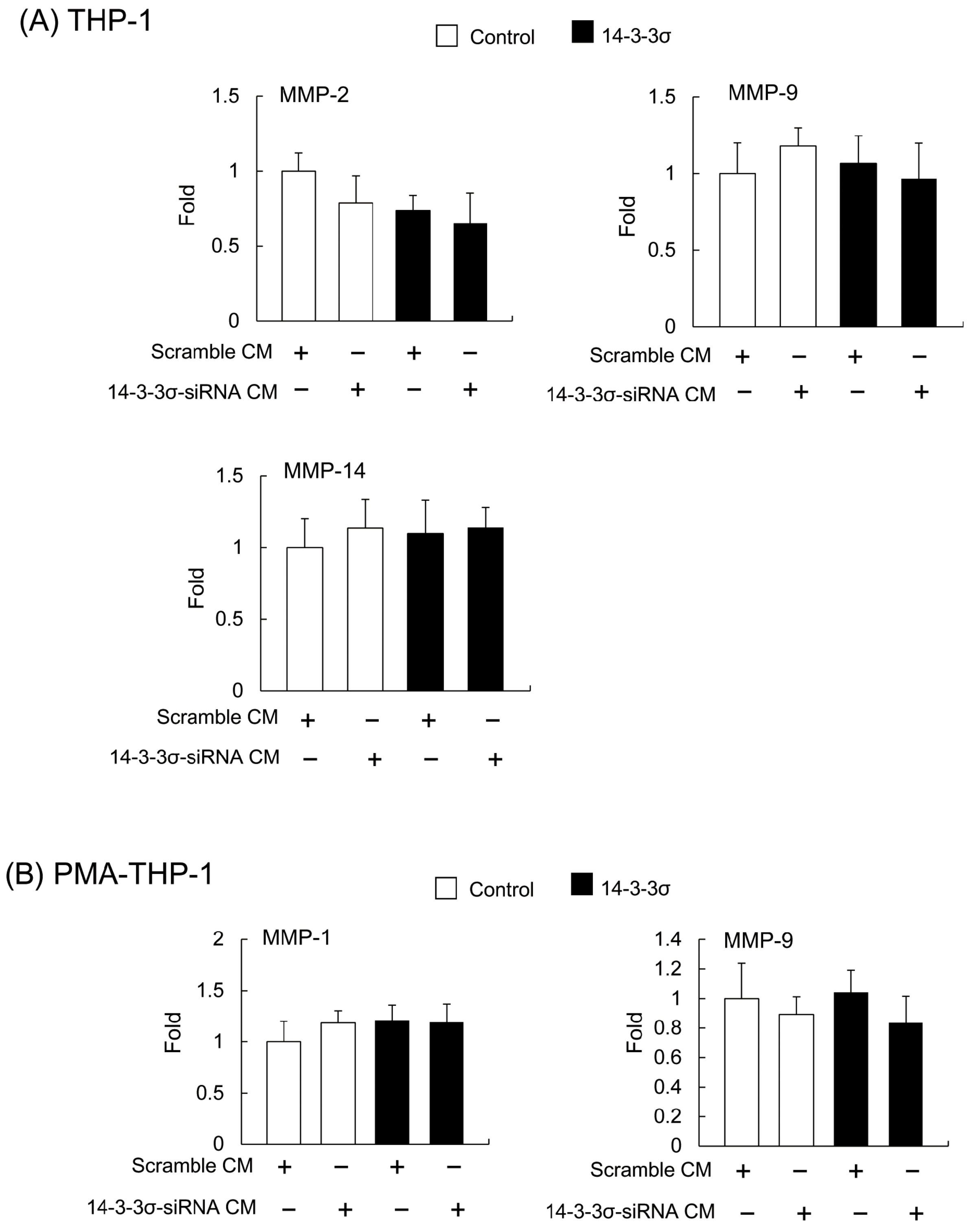

Supplementary Figure S3: Effect of 14-3-3σ silencing on 14-3-3σ-CM induced MMPs expression. A. THP-1 cells were incubated with CM and expression of MMPs was determined by Q-PCR. **B.** PMA-THP-1 cells were incubated with CM and expression of MMPs was determined by Q-PCR. These results are from three independent experiments. Scale bars: mean ± SD.

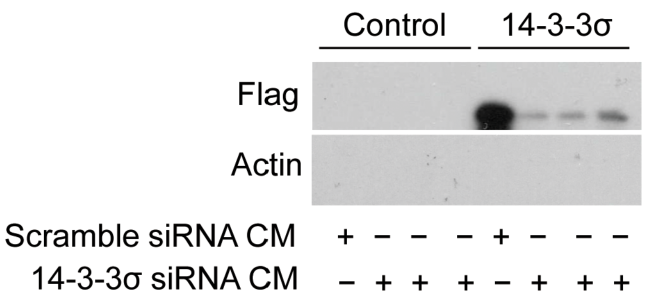

Supplementary Figure S4: Expression of 14-3-3σ in CM from 14-3-3σ stable cells was abolished by siRNA. 14-3-3σ stable and control cells were transfected with scramble or three different 14-3-3σ siRNA sequences for 48h. CM was harvested and expression of 14-3-3σ was determined by Western blot analysis of Flag antibody. Actin was used as loading control.

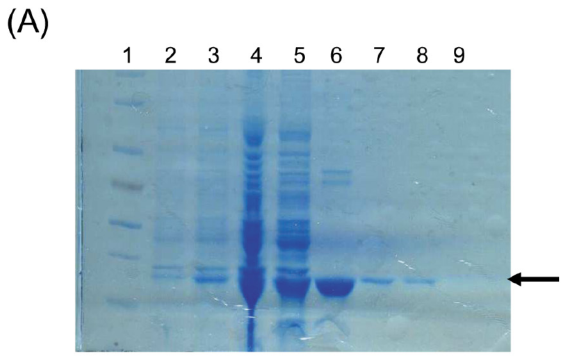

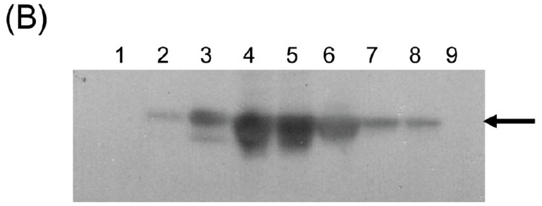

Supplementary Figure S5: Purification and identification of recombinant 14-3-3σ (r14-3-3σ) proteins. cDNA of 14-3-3σ was amplified and cloned into a BL21/pET28a vector. Expression of r14-3-3σ was induced by IPTG (1 mM, 4h) followed by purification. **A.** SDS-PAGE analysis of r14-3-3σ protein. Samples were analyzed on 4-12% SDS polyacrylamide gel follow by coomassie blue staining. **B.** The expression of r14-3-3σ was confirmed by Western blot analysis. Arrow indicates r14-3-3σ. Lane 1, protein marker; Lane 2, uninduced cell lysate of BL21/pET28a-14-3-3σ; Lane 3, IPTG induced BL21(DE3)/pET28a-14-3-3σ; Lane 4, post-sonication sample were collected after 8 minutes, supernatant of the bacterial lysate; Lane 5, post-sonication samples were collected after 12 minutes, supernatant of the bacterial lysate; Lane 6, eluted BL21(DE3)/pET28a-14-3-3σ of the fraction 1; Lane 7, eluted BL21(DE3)/pET28a-14-3-3σ of the fraction 2; Lane 8, eluted BL21(DE3)/pET28a-14-3-3σ of the fraction 3; Lane 9, wash buffer was collected after the final elution step.

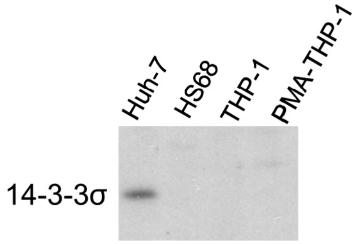

Supplementary Figure S6: Endogenous protein levels of 14-3-3σ in Huh-7, HS68, THP-1 and PMA-THP-1 cells were analyzed by Western blot analysis.

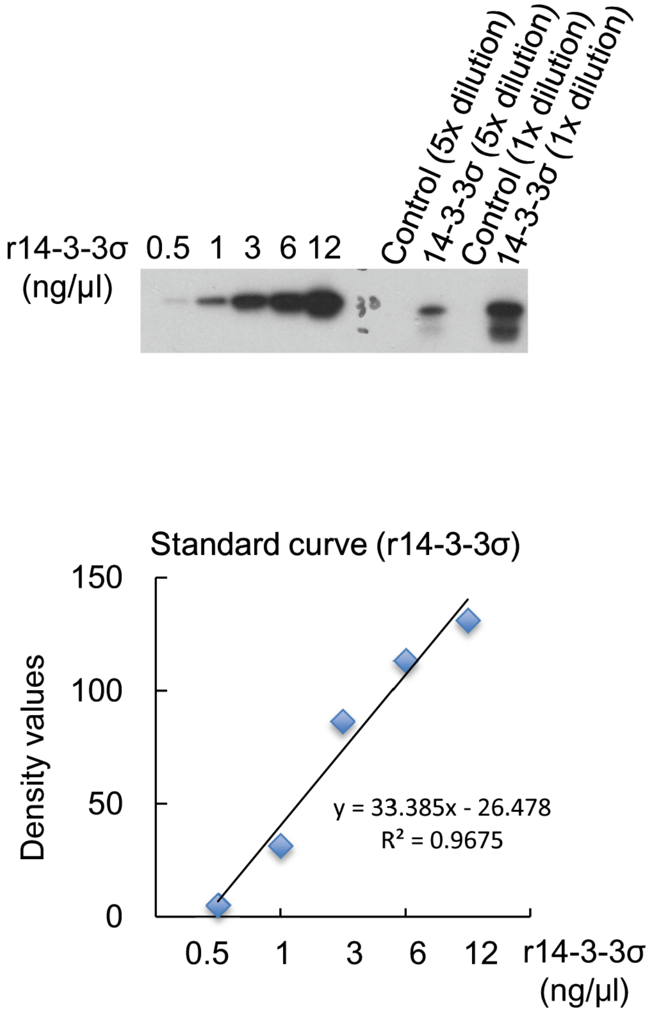

Supplementary Figure S7: Quantification of 14-3-3σ level in CM. The expression of 14-3-3σ in CM of control and 14-3-3σ stable cells (1X and 5X dilution, 20 μl per lane) was determined by Western blot analysis (upper panel). The r14-3-3σ proteins (0.5, 1, 3, 6 and 12 ng/μl) were loading as control for calibration of protein concentrations. The quantification of 14-3-3σ level in CM was estimated by densitometry according to the detection of the concentration of r14-3-3σ as standard curve (lower panel).

Supplementary Table S1: Oligonucleotide sequences for Q-PCR

Gene	Primers Sequences
MMP-1	Fw 5′- AGCTAGCTCAGGATGACCTTGATG -3′
	Rv 5′- GCCGATGGGCTGGACAG -3′
MMP-2	Fw 5′- TGCTGGAGACAAATTCTGGAGATAC -3′
	Rv 5′- ACTTCACGCTCTTCAGACTTTGG -3′
MMP-9	Fw 5′- AGGACGGCAATGCTGATG -3′
	Rv 5′- TCGTAGTTGGCGGTGGTG -3′
MMP-12	Fw 5′- CATGAACCGTGAGGATGTTGA -3′
	Rv 5′- GCATGGGCTAGGATTCCACC -3′
MMP-14	Fw 5′- CGAGGTGCCCTATGCCTAC -3′
	Rv 5′ - CTCGGCAGAGTCAAAGTGG -3′
ANPEP (APN)	Fw 5′ - CTGTGAGCCAGTCTAGTTCCTGAT -3′
	Rv 5′ - CATCGAGAGCTTCTGCTCATCT -3′

Supplementary Table S2: Oligonucleotide sequences of small interfering RNAs

Accession no.	Gene name	siRNA primer sequences	Concentration (nm)
NM_006142.3	SFN (14-3-3σ)	Sequence 1	40
		Fw 5′- UCUCAGUAGCCUAUAAGAACGUGGU -3′	
		Rv 5′- AGAGUCAUCGGAUAUUCUUGCACCA -3′	
		Sequence 2	
		Fw 5′-CCGUCUUCCACUACGAGAUCGCCAA-3′	
		RV 5′-GGCAGAAGGUGAUGCUCUAGCGGUU-3′	
		Sequence 3	
		Fw 5′-GCGCAUCAUUGACUCAGCCCGGUCA-3′	
		Rv 5′-CGCGUAGUAACUGAGUCGGGCCAGU-3′	
NM_001150.2	ANPEP (APN)	Sequence 1	
		Fw 5′-GAUUAUGGUGGUGGCUCGUUCUCCU-3′	60
		Rv 3′-CUAAUACCACCACCGAGCAAGAGGA-5′	
		Sequence 2	
		Fw 5′-GGCCACCACCUUGGACCAAAGUAAA -3′	
		Rv 3′-CCGGUGGUGGAACCUGGUUUCAUUU-5′	
		Sequence 3	
		Fw 5′-CCAACACGCUGAAACCCGAUUCCUA-3′	
		Rv 3′- GGUUGUGCGACUUUGGGCUAAGGAU-5′	
